# Utility of *CD-138* negative fraction for chromosome analysis in Plasma Cell Dyscrasias (PCD): a novel approach

**DOI:** 10.1186/1755-8166-7-S1-O5

**Published:** 2014-01-21

**Authors:** Gopalrao Velagaleti, Christina Mendiola, Gihan Mohamed, William Ehman, Vinaya Noronha, Veronica Ortega

**Affiliations:** 1 Department of Pathology, University of Texas Health Science Center, 7703 Floyd Curl Drive, San Antonio, TX, USA

## Background

FISH analysis is superior to chromosome analysis in detecting important prognostic genetic abnormalities in PCD. However, its sensitivity is hampered due to paucity of plasma cells in whole bone marrow and often shows false-negative results when frequency of abnormal cells is below the cut-off values. Studies have shown that the abnormality detection rate in enriched plasma cells (EPC) is greater than unselected plasma cells, but purification techniques are limiting to only FISH when bone marrow volumes are inadequate. The inability to perform chromosome analysis may compromise patient care since chromosome analysis is equally important for detecting non-plasma cell related abnormalities, such as secondary myelodysplastic syndrome. To resolve this critical issue and optimize limited quantity received, we designed a study where an immuno-magnetic CD138 enriched positive selection of plasma cells was used for FISH while the negative selection was used to retrieve the remaining cellular components (RCC) for chromosome analysis. After validating this approach in a pilot study, we implemented this strategy in 2012 for routine clinical diagnosis in patients with PCD. When there was adequate sample volume available, both whole bone marrow and RCC were used for chromosome analysis.

## Results

We found 100% success rate for chromosome analysis using RCC. Identical PCD related genetic abnormalities were observed by both chromosome analysis using whole bone marrow and EPC FISH in 4.2% (4/96) cases while 8.3% (8/96) cases showed discordant results. Population variants such as loss of Y chromosome were observed in 6.3% (6/96) cases. Karyotypic abnormalities of myeloid origin or population variants were found both in whole bone marrow and RCC cultures. Karyotypes with PCD related aberrations were seen only in whole bone marrow cultures in 37.5% of the cases (3/8) while the remaining 62.5% (5/8) of cases showed them in both whole bone marrow and RCC cultures. All PCD related aberrations showed concordant EPC FISH results.

## Conclusions

Our results confirm the feasibility of retrieving RCC from the CD138 negative fraction, and prove to be an innovative strategy for performing chromosome analysis on PCD patients with insufficient sample volumes.

